# Two novel microRNAs and their association with absolute blood pressure parameters in an urban South African community

**DOI:** 10.1007/s11033-021-06304-1

**Published:** 2021-03-23

**Authors:** Don M. Matshazi, Cecil J. Weale, Rajiv T. Erasmus, Andre P. Kengne, Saarah F. G. Davids, Shanel Raghubeer, Glenda M. Davison, Tandi E. Matsha

**Affiliations:** 1 SAMRC/CPUT/Cardiometabolic Health Research Unit, Department of Biomedical Sciences, Faculty of Health and Wellness Sciences, Cape Peninsula University of Technology, 1 Symphony Way, Bellville, PO Box 1906, Cape Town, 7530 South Africa; 2 Division of Chemical Pathology, Faculty of Health Sciences, National Health Laboratory Service (NHLS), University of Stellenbosch, Cape Town, South Africa; 3 Non-Communicable Diseases Research Unit, South African Medical Research Council, Cape Town, South Africa; 4 Department of Medicine, University of Cape Town, Cape Town, South Africa

**Keywords:** Hypertension, Novel microRNA, Blood pressure, Africa, RT-qPCR, MiRNA sequencing

## Abstract

MicroRNAs are important in development of disease, and description of novel microRNAs adds to the pool of microRNAs that can be targeted for diagnostic and therapeutic purposes in disease. Herein, we aimed to describe novel microRNAs in a normotensive and hypertensive African population and relate their expression to blood pressure parameters and hypertension status. Previous work using next-generation sequencing showed differential expression of two novel microRNAs in the blood of normotensives and hypertensives. Herein, we have investigated these novel microRNAs by quantitative reverse transcription polymerase chain reaction in a cohort of 881 participants in this study. The relationship between the novel microRNAs and systolic and diastolic blood pressure as well as mean arterial pressure was also investigated. Age and sex-adjusted Spearman’s correlations were used to assess the relationship between microRNAs and cardiovascular risk profile variables whilst multivariable logistic regression models were used to assess the association of microRNAs with screen-detected and known hypertension. The novel microRNAs (miR-novel-chr1_36178 and miR-novel-chr15_18383) were significantly dysregulated by hypertension status. The expression of miR-novel-chr1_36178 differed according to sex, correlated with mean arterial pressure and systolic and diastolic blood pressure at higher levels of expression and was associated with screen-detected hypertension. The association of miR-novel-chr1_36178 expression with mean arterial pressure and systolic and diastolic blood pressure, as well as its dysregulation according to hypertension status suggests its possible utility as a biomarker target for hypertension diagnosis and/or therapeutics. Furthermore, its association with screen detected hypertension and dose-response relationship with blood pressure suggests it may be used to identify and monitor individuals at risk of hypertension.

**Supplementary Information:**

The online version contains supplementary material available at 10.1007/s11033-021-06304-1.

## Introduction

Ever since the discovery of the first non-coding ribonucleic acid (RNA) close to 30 years ago [1], more than 2000 mature human microRNAs (miRNAs) have since been described [2]. Using small RNA sequencing techniques, epigenome studies continue to describe novel miRNAs in various body tissues and disease conditions [3, 4]. These miRNAs are 18–22 nucleotides long [5] and are involved in the regulation of gene expression at the post transcription level, either through targeted messenger RNA (mRNA) degradation or inhibition of translation [3, 6]. As such, they have a crucial role in maintaining the homeostatic balance of the human body’s processes and dysregulations in their expression may be related to disease processes [7, 8].

An individual’s phenotype is strongly influenced by the complex interplay between their genetic makeup and the environment they inhabit [9]. Identifying variations in the genetic (particularly the epigenome) makeup of different populations may help us understand and explain why different populations respond to the same disease and/or medications in a different manner. As the impact of miRNAs in the world of science and disease continues to grow steadily, the conduction of studies on miRNAs in disease, including hypertension (HPT), has gained considered impetus in high income Asian [10–12] and European [13] countries with potentially interesting therapeutic implications [14]. However, there is a paucity of studies focusing on miRNAs and HPT in the African population, despite its well-described genetic diversity [15] and steadily increasing HPT prevalence [16].

Herein, we present two previously undescribed microRNAs exhibiting distinct expression patterns in a hypertensive African population when compared to normotensives from the same community. We further interrogated the relationship between the expression of these novel miRNAs and blood pressure parameters (systolic blood pressure (SBP), diastolic blood pressure (DBP) and mean arterial pressure (MAP)). Establishment of specific epigenomic signatures with clinical parameters of disease may offer alternative diagnostic and prognostic avenues, in addition to the potential development of effective, population-targeted therapeutic measures, instead of blanket approaches for genetically diverse populations.

## Materials and methods

### Study design and procedures

This was a cross-sectional study composed of male and female participants from the Vascular and Metabolic Health (VMH) study, an extension of the Cape Town Bellville South study, previously described [17]. Data collection and procedures have been reported previously [4]. Briefly, each participant underwent anthropometric measurements and these were reported as the average of three separate readings. Body Mass Index (BMI) was calculated as weight per square meter (kg/m^2^) where kg is a participant’s weight in kilograms and m^2^ is the square of their height in metres. Blood pressure (BP) was measured according to the World Health Organisation (WHO) guidelines [18], using a semi-automatic digital BP monitor (Omron M6 comfort-preformed cuff blood pressure monitor, China) on the right arm in a sitting position and at rest for at least 10 min. Three BP readings were taken at three-minute intervals and the lowest SBP and corresponding DBP values were used. Participants were grouped into two categories based on BP measurement of 140/90 mm Hg as screen-detected HPT and normal BP measurement as normotensive.

Various biochemical parameters were measured in an ISO 15,189 accredited pathology practice (PathCare Reference Laboratory, Cape Town, South Africa) using different analytical methods as follows: glycated haemoglobin (HbA1c) by High Performance Liquid Chromatography (BioRad Variant Turbo, BioRad, Hercules, CA, USA); serum insulin by a paramagnetic particle chemiluminescence assay (Beckman DXI, Beckman Coulter, South Africa); serum cotinine by Competitive Chemiluminescent **(**Immulite 2000, Siemens, Munich, Germany); plasma glucose by enzymatic hexokinase method (Beckman AU, Beckman Coulter, Brea, CA, USA); total cholesterol (TC); high density lipoprotein cholesterol (HDL-c) by enzymatic immunoinhibition—end point (Beckman AU, Beckman Coulter, Brea, CA, USA); triglycerides (TG) by glycerol phosphate oxidase–peroxidase, end point (Beckman AU, Beckman Coulter, Brea, CA, USA); low density lipoprotein cholesterol (LDL) by enzymatic selective protection—end point (Beckman AU, Beckman Coulter, Brea, CA, USA); and ultrasensitive C-reactive protein (CRP) by Latex Particle Immunoturbidimetry (Beckman AU, Beckman Coulter, Brea, CA, USA). These analyses were conducted at an ISO 15,189 accredited pathology practice (PathCare Reference Laboratory, Cape Town, South Africa). Blood samples for miRNA expression analysis were collected in Tempus Blood RNA tubes (ThermoFisher Scientific, Waltham, MA, USA) and stored at − 80 °C for total RNA extraction and quantitative reverse transcription polymerase chain reaction (RT-qPCR) analysis.

### RNA isolation

Total RNA, including miRNA, was isolated from 3 mL of whole blood using the MagMax Total RNA isolation kit (ThermoFisher Scientific) according to manufacturer’s instructions, with the nucleic acid washing and elution steps conducted on the Kingfisher Flex system. The concentration and purity of each total RNA extract was determined using a NanoDrop One spectrophotometer and total RNA extracts with 260/280 values between 1.8 and 2.0, and concentrations greater than 20 ng/µl were used for RT-qPCR.

### MicroRNA sequencing using next generation sequencing

This was conducted on samples from a cohort of 48 age-matched, normotensive, screen-detected hypertensive and known hypertensive female participants. Small RNA library construction, deep sequencing, and data processing were performed at Arraystar Inc., Rockville, USA as previously described [4]. Briefly, the total RNA of each sample was used to prepare the miRNA sequencing library as follows:1) 3′-adapter ligation with T4 RNA ligase 2 (truncated); 2) 5′-adapter ligation with T4 RNA ligase; 3) complementary deoxyribonucleic acid (cDNA) synthesis with RT primer; 4) PCR amplification; 5) extraction and purification of ~ 130–150 bp PCR amplified fragments (correspond to ~ 15–35 nt small RNAs) from the polyacrylamide gel electrophoresis (PAGE) gel. Completed libraries were quantified using the Agilent 2100 Bioanalyzer, followed by denaturation of DNA fragments using 0.1 M sodium hydroxide to generate single-stranded DNA molecules, which were then captured on Illumina flow cells, amplified in situ, and sequenced on the Illumina HiSeq system for 51 cycles according to the manufacturer’s instructions. Raw sequences were generated as clean reads from the Illumina HiSeq using real-time base calling and quality filtering. Adaptor sequences were removed from clean reads that passed the quality filter to produce trimmed reads of length ≥ 15 nucleotides. Using NovoAlign software, the trimmed reads were aligned to the human pre-miRNA in miRBase 21. The miRNA expression levels were measured and normalized as transcripts per million (TPM) of total aligned miRNA reads. MicroRNAs with fold changes ≥ 1.3, and *p*-values ≤ 0.1 were selected as the differentially expressed miRNAs. Novel miRNAs were predicted using miRDeep.

### Quantitative reverse transcription PCR

RT-qPCR was performed to determine the novel miRNA expression levels in an independent sample of 881 normotensive, screen-detected hypertensive and known hypertensive male and female participants, as determined by next generation sequencing. Briefly, using the TaqMan Advanced miRNA cDNA synthesis kit (Applied Biosystems, ThermoFisher Scientific, Waltham, MA, USA), 2 µl of total RNA was converted into cDNA through sequential conduction of poly-A tailing, adaptor ligation, reverse transcription and miR-Amp steps as per the manufacturer’s recommendation. The miRNA expression levels were then determined using TaqMan miRNA Assay primers (Applied Biosystems, ThermoFisher Scientific, Waltham, MA, USA) on the QuantStudio 7 Flex real-time PCR instrument (Life Technologies, Carlsbad, CA, USA). In order to determine relative miRNA expression in each sample, the 2^−ΔCt^ method was used whilst the 2^−ΔΔCt^ method was used to compute fold change differences in miRNA expression between the study groups using miR-16-5p (ThermoFisher Scientific, Waltham, MA, USA) as the endogenous control [19].

### Statistical Analysis

Data analysis was performed using Statistical Product and Service Solutions (SPSS) v.26 software (IBM Corp, USA). Normally distributed variables are reported as count (and percentages), mean (and standard deviation) whilst asymmetrically distributed variables are reported as median (25th-75th percentiles). Comparisons of median and mean baseline characteristics across BP groups were done using the Kruskal–Wallis test and analysis of variance (ANOVA) respectively. Spearman’s correlations, adjusted for age and sex, were used to assess the relationship between miRNAs and other cardiovascular risk profile variables whilst multivariable logistic regression models were used to assess the association of miRNAs with screen-detected HPT and known HPT with crude and adjusted odds ratio (OR). A *p*-value less than 0.05 signified statistically significant findings.

## Results

### Cohort description

The study was made up of 598 (67.9%) female and 283 (32.1%) male participants with a mean age of 42.6 years. BP parameters (SBP, DBP and MAP) did not show any significant difference by sex (*p* ≥ 0.085). The expression of miR-novel-chr1_36178 significantly differed according to sex (*p* = 0.002), with greater expression in males compared to females. Other significant variations based on sex are shown in Table 1. Briefly, anthropometric, glycaemic and lipid indices were significantly raised in women, *p* ≤ 0.043 whilst gamma glutamyltransferase (GGT) was reduced, *p* = 0.002.

**Table 1:** Characteristics of participants that took part in the study

	All	Female	Male	*p*-value
	*n* = 881 mean ± SD	*n* = 598 mean ± SD	*n* = 283 mean ± SD	
miR-novel-chr1_36178 (2^−ΔCt^)*	0.6594 (0.2471;1.5976)	0.569 (0.2205;1.4008)	0.8583 (0.2986;1.9741)	0.002
miR-novel-chr15_18383 (2^−ΔCt^)*	0.0013 (0.0004;0.0037)	0.0013 (0.0004;0.0037)	0.0013 (0.0005;0.0038)	0.848
Age (years)	42.6 ± 14.3	42.9 ± 14.4	42.0 ± 14.2	0.415
Body mass index	26.8 ± 7.6	28.6 ± 8.0	23.0 ± 5.3	< 0.001
Waist circumference (cm)	86.8 ± 16.0	89.3 ± 16.3	81.5 ± 13.8	< 0.001
Hip circumference (cm)	99.6 ± 15.6	103.3 ± 15.8	91.6 ± 11.6	< 0.001
Waist to hip ratio*	0.87 (0.82;0.92)	0.86 (0.81;0.92)	0.88 (0.84;0.94)	< 0.001
Systolic blood pressure (mmHg)	128.1 ± 23.1	127.8 ± 23.2	128.6 ± 22.8	0.663
Diastolic blood pressure (mmHg)	82.1 ± 14.2	82.6 ± 13.5	80.9 ± 15.3	0.085
Mean arterial pressure	97.4 ± 16.1	97.7 ± 15.7	96.8 ± 17.0	0.422
Fasting blood glucose (mmol/L)*	4.7 (4.4;5.2)	4.7 (4.5;5.2)	4.7 (4.3;5.2)	0.043
2-h glucose (mmol/L)*	5.5 (4.5;6.9)	5.9 (4.9;7.2)	4.7 (3.9;6)	< 0.001
HbA1c (%)*	5.6 (5.3;5.9)	5.6 (5.3 5.9)	5.5 (5.3;5.8)	0.046
HbA1c (mmol/mol)*	37.7 (34.4;41.0)	37.7 (34.4;41.0)	36.6 (34.4;39.9)	0.046
Fasting insulin (mIU/L)*	5.8 (3.6;9.1)	6.6 (4.2;9.6)	4.3 (2.7;7.3)	< 0.001
2-h insulin (mIU/L)*	30.8 (15.7;56.8)	38.4 (22.0;70.9)	18.4 (8.4;36.0)	< 0.001
Triglycerides (mmol/L)*	1.07 (0.78;1.52)	1.05 (0.76;1.48)	1.11 (0.81;1.63)	0.093
Total cholesterol (mmol/L)	4.97 ± 1.14	5.1 ± 1.15	4.69 ± 1.07	< 0.001
HDL-cholesterol (mmol/L)	1.35 ± 0.41	1.38 ± 0.41	1.29 ± 0.39	0.002
LDL-cholesterol (mmol/L)	3.0 ± 0.1	3.2 ± 0.1	2.8 ± 0.9	< 0.001
C-reactive protein (mg/L)*	3.0 (1.2;7.8)	3.5 (1.4;8.5)	2.3 (1.0;6.0)	0.008
Gamma GT-S* (IU/L)*	27 (19;42)	26 (18;40)	29 (21;47)	0.002

Values presented as mean ± SD unless marked with an asterisk*, in which case the median and (25^th^–75th percentiles) are reported. The Kruskal–Wallis test and analysis of variance (ANOVA) were used to compare the median and mean baseline characteristics respectively between sex

*SD* standard deviation

### Whole miRNA sequencing

Two novel miRNAs (miR-novel-chr15_18383 and miR-novel-chr1_36178) were discovered using next generation sequencing techniques and their characteristics are described in Table 2. miR-novel-chr15_18383 is transcribed from a gene located on chromosome 15, whilst miR-novel-chr1_36178 is from a gene located on chromosome 1. Their expression differed significantly according to HPT status and whilst the expression of miR-novel-chr1_36178 was significantly higher in known hypertensives on therapy compared to the normotensives (fold difference = 1.5), the expression of miR-novel-chr15_18383 was significantly higher in known hypertensives on therapy compared to screen-detected hypertensives (fold difference = 1.3).

**Table 2:** Description of significantly differentially expressed novel microRNAs discovered by next generation sequencing

Mature ID	Pre-miRNA accession ID	Mature seed sequence	Mature length	Mature sequence
hsa-miR-novel-chr1_36178	MYNO2414	UCCAGC	17	CUCCAGCCUGGGCAACA
hsa-miR-novel-chr15_18383	MYNO1379	GCUCCC	22	UGCUCCCCCUCCCUUCCUGGGA

### Linear correlations

As shown in Table 3, there was a weak, positive correlation between the expression of miR-novel-chr15_18383 and miR-novel-chr1_36178 (*r* = 0.48, *p* < 0.001). There was no correlation between the expression of miR-novel-chr15_18383 and BP parameters. Interestingly, although very weak, there was a positive correlation between miR-novel-chr1_36178 expression and both DBP and MAP (*r* ≤ 0.08, *p* ≤ 0.024).

**Table 3:** Spearman correlations for blood pressure parameters and novel miRNA expression

	miR-novel-chr1_36178 (2^−ΔCt^)		miR-novel-chr15_18383 (2^−ΔCt^)	
	*r*	*p*-value	r	*p*-value
miR-novel-chr1_36178 (2^−ΔCt^)	1.000		0.477	< 0.001
miR-novel-chr15_18383 (2^−ΔCt^)	0.477	< 0.001	1.000	
Systolic blood pressure (mmHg)	0.064	0.060	0.026	0.453
Diastolic blood pressure (mmHg)	0.082	0.016	0.048	0.162
Mean arterial pressure (mmHg)	0.076	0.024	0.045	0.195

### Linear regression models

Table 4 shows models used to predict absolute BP values based on the expression levels of the two novel miRNAs. For models 1 and 2, the variables were measured at the continuous level. MicroRNA expression data values were log-transformed before analysis to cater for skewness. Whilst expression levels of miR-novel-chr15_18383 could not significantly predict the SBP, DBP and MAP as shown in Model 2, there was statistical significance in the expression of miR-novel-chr1_36178 and prediction of SBP, DBP and MAP. For every 1-unit increase in the expression of miR-novel-chr1_36178, there is a 0.03 increase in the SBP (*p* = 0.003), a 0,01 increase in the DBP (*p* = 0.035) and 0.02 increase in the MAP (*p* = 0.008).

**Table 4:** Age and sex adjusted linear regression models for determinants of absolute BP levels and Mean Arterial Pressure^#^

	SBP		DBP		MAP	
	B (95% CI)	*p*-value	B (95% CI)	*p*-value	B (95% CI)	*p*-value
Model 1						
miR-novel-chr1_36178	0.03 (0.01; 0.05)	0.003	0.01 (0; 0.03)	0.035	0.02 (0; 0.03)	0.008
Model 2						
miR-novel-chr15_18383	0.02 (0; 0.04)	0.123	0.01 (0; 0.02)	0.073	0.01 (0; 0.03)	0.071
Model 3						
miR-novel-chr1_36178 Q2	1.91 (− 2.39; 6.22)	0.382	− 0.37 (− 3.21; 2.47)	0.800	0.39 (− 2.71; 3.5)	0.803
miR-novel-chr1_36178 Q3	3.09 (− 1.2; 7.39)	0.158	0.29 (− 2.54; 3.13)	0.839	1.23 (− 1.88; 4.33)	0.438
miR-novel-chr1_36178 Q4	2.24 (− 2.08; 6.55)	0.309	0.33 (− 2.52; 3.17)	0.821	0.96 (− 2.15; 4.08)	0.544
miR-novel-chr1_36178 Q5	6.99 (2.66; 11.32)	0.002	4.2 (1.35; 7.06)	0.004	5.13 (2.01; 8.26)	0.001
Model 4						
miR-novel-chr15_18383 Q2	− 3.91 − -8.39; 0.56)	0.086	− 0.45 (− 3.39; 2.49)	0.766	− 1.6 (− 4.83; 1.62)	0.330
miR-novel-chr15_18383 Q3	0.97 (− 3.53; 5.46)	0.673	0.89 (− 2.06; 3.84)	0.554	0.92 (− 2.32; 4.16)	0.579
miR-novel-chr15_18383 Q4	0.15 (− 4.32; 4.62)	0.947	1.84 (− 1.1; 4.78)	0.219	1.28 (− 1.94; 4.5)	0.437
miR-novel-chr15_18383 Q5	0.94 (− 3.54; 5.42)	0.680	1.27 (− 1.67; 4.21)	0.397	1.16 (− 2.07; 4.39)	0.481

^#^Linear regression models showing the relationship between SBP, DBP and MAP. In model 1 and 2, log-transformed variables were used to cater for the skewness in miRNA expression data. In model 3 and 4, the dose response for each miRNA was determined by dividing the relative miRNA expression into five quintiles, with Quintile 1 (Q1) as the reference

A dose response assessment was done to determine if the effect of the novel miRNAs on BP variables was dependant on the expression levels of the miRNAs. The miRNA expression data was divided into five quantiles (Q1-5), with Q1 being used as the reference. Although a significant relationship between the expression of miR-novel-chr1_36178 and BP variables was shown in Model 1, the significance of that relationship remained only when the miRNA was expressed at levels falling in quantile 5 (Q5) (*p* ≤ 0.004). Findings in Model 4 concurred with Model 2 findings, indicating that even when expressed at high levels, miR-novel-chr15_18383 had no significant relationship with SBP, DBP and MAP.

### Regression analysis

Age and sex adjusted OR were calculated to quantify the strength of the relationship between miRNA expression and screen-detected HPT, using the normotensives as a reference. As shown in Table 5, the expression of miR-novel-chr1_36178 and miR-novel-chr15_18383 was significantly associated with screen-detected HPT, OR = 1.36 (95% Confidence Interval (CI) 1.08–1.70), *p* = 0.008 and OR = 1.31 (95% CI 1.05–1.63), *p* = 0.016. As for the dose response analysis, a significant association between the expression of miR-novel-chr1_36178 and the presence of screen-detected HPT was seen only when the miRNA was expressed at the highest level (Q5), OR = 2.13 (95% CI 1.32–3.45), *p* = 0.002. In contrast, there was no dose dependant association between the expression of miR-novel-chr15_18383 and the presence of screen-detected HPT, even at Q5 levels.

**Table 5:** Age and sex adjusted odds ratios (with 95% confidence intervals) for determinants of screen-detected hypertension

	Normotensive vs Screen-detected hypertension		
	Odds ratio	95% CI	p-value
Model 1			
miR-novel-chr1_36178	1.36	(1.08; 1.70)	0.008
Model 2			
miR-novel-chr15_18383	1.31	(1.05; 1.63)	0.016
Model 3			
miR-novel-chr1_36178 Q1	1		
miR-novel-chr1_36178 Q2	1.32	(0.82; 2.14)	0.255
miR-novel-chr1_36178 Q3	1.52	(0.94; 2.46)	0.086
miR-novel-chr1_36178 Q4	1.5	(0.93; 2.43)	0.098
miR-novel-chr1_36178 Q5	2.13	(1.32; 3.45)	0.002
Model 4			
miR-novel-chr15_18383 Q1	1		
miR-novel-chr15_18383 Q2	1.04	(0.64; 1.70)	0.871
miR-novel-chr15_18383 Q3	1.38	(0.85; 2.24)	0.188
miR-novel-chr15_18383 Q4	1.55	(0.96; 2.51)	0.072
miR-novel-chr15_18383 Q5	1.37	(0.84; 2.22)	0.208

For model 1 and 2, log-transformed variables were used to cater for skewed miRNA expression data. In model 3 and 4, the dose response for each of the novel miRNAs was determined by dividing the miRNA expression into five quintiles, with Quantile 1 (Q1) as the reference

## Discussion

Whole genome sequencing showed the differential expression of two novel microRNAs (miR-novel-chr1_36178 and miR-novel-chr15_18383) of length 17 and 22 nucleotides respectively, in peripheral blood from normotensive and hypertensive individuals. These findings were confirmed in a larger, independent sample by RT-qPCR. The expression of miR-novel-chr15_18383 had no association with sex and did not correlate with any of the three BP parameters at lower or higher levels of expression. In contrast, the expression of miR-novel-chr1_36178 significantly differed according to sex. At lower levels of expression, the miRNA had no association with screen-detected HPT or SBP, DBP and MAP. Interestingly, when expressed at greater levels, the miRNA was associated with all three BP parameters and the odds of screen-detected HPT in the presence of miR-novel-chr1_36178 doubled.

Whilst the influence of miRNAs on gene regulation has been previously described [20, 21], the effects of the extent to which a miRNA is expressed relative to its function remains an understudied aspect. However, Shu and colleagues demonstrated the dose-dependant function of a panel of miRNAs (let-7a-7f and the miR-17–92 cluster) and postulated that target mRNA selection may not only be dependant on sequence homology as commonly described, but also on the endogenous expression levels of the particular miRNA [22]. It is plausible that the miR-novel-chr1_36178 described in our study, also exhibits this dose-dependant characteristic in BP regulation.

Whilst the relationship between expression of these two novel miRNAs and BP variables or HPT status cannot be directly compared to similar studies (as our findings are novel), differential expression of miRNAs in HPT has been previously reported [5, 13] and expression of miRNAs has also been related to BP parameters. For instance, one study showed a significant correlation between SBP and the expression of miR-21, miR-126 and miR-34a has been reported, whilst miR-146a expression was correlated with SBP and DBP [23]. An elevated expression of miR-21 in hypertensives compared to normotensives has previously been reported and there was a positive correlation between miR-21 expression and clinical and ambulatory SBP [24], suggestive of a potential relationship between the miRNA and the regulation of BP. The maintenance of vascular integrity is one of the important processes carried out by endothelial cells [25] and miR-126, one of the miRNAs showing a differential expression pattern in hypertensives and correlated to BP parameters in a study by Hijmans and colleagues, plays an integral role in this. As such, changes in the concentrations of miRNAs in hypertensives may represent an increased risk of disease in the vasculature of older people, increasing the possibility of a cardiovascular event.

In conclusion, miR-novel-chr1_36178 showed significant dysregulation in hypertensives and its expression at higher levels, was related to SBP, DBP and MAP. As such, it warrants further study to understand its dose dependant relationship with BP parameters and the possible role it plays in the pathogenesis of HPT. As it is a previously unreported miRNA, it has to be further characterised to understand its physiology, target networks and pathways in the body. This may shed more light on how its expression impacts BP regulation.

The study had some limitations. Not much is known about these novel miRNAs and as such, findings could not be directly compared to other studies at this stage. However, the discovery of these novel miRNAs adds to the pool of miRNAs that can be targeted for study in HPT and cardiovascular diseases.

## Supplementary Information

Below is the link to the electronic supplementary material.

Supplementary file1 (DOCX 13 kb)

## Data Availability

The datasets used and/or analysed during the current study are available from the National Center for Biotechnology Information (NCBI) Seqeunce Read Archive on the following link: https://www.ncbi.nlm.nih.gov/sra/PRJNA680302

## Abbreviations

ANOVA: Analysis of variance
BMI: Body mass index
BP: Blood pressure
cDNA: Complementary DNA
CI: Confidence interval
CRP: C-Reactive Protein
DBP: Diastolic blood pressure
DNA: Deoxyribonucelic acid
GGT: Gamma glutamyltransferase
HbA1c: Glycated haemoglobin
HDL-c: High density lipoprotein cholesterol
HPT: Hypertension
LDL: Low density lipoprotein
MAP: Mean arterial pressure
miRNA: MicroRNA
mRNA: Messenger RNA
NCBI: National center for biotechnology information
OR: Odds ratio
RNA: Ribonucleic acid
RT-qPCR: Quantitative reverse transcription polymerase chain reaction
SAMRC: South African medical research council
SBP: Systolic blood pressure
SPSS: Statistical product and service solutions
TC: Total cholesterol
TG: Triglycerides
TPM: Transcripts per million
VMH: Vascular and metabolic health
WHO: World health organisation
