# Supplementary material for: Two novel microRNAs and their association with absolute blood pressure parameters in an urban South African community
Source: Mol Biol Rep. 2021 Mar 23;48(3):2553–60. doi: 10.1007/s11033-021-06304-1 (PMC8060202; doi:10.1007/s11033-021-06304-1)
Supplement: Supplementary file 1 — Supplementary file1 (DOCX 13 kb) [file 11033_2021_6304_MOESM1_ESM.docx]

**Supplementary Table 1.** Description of significantly differentially expressed novel microRNAs discovered by next generation sequencing

| **Mature ID** | **Pre-miRNA accession ID** | **Mature seed sequence** | **Mature length** | **Mature sequence** | **TPM** | | |
| --- | --- | --- | --- | --- | --- | --- | --- |
|  | | | | | **Known HPT** | **Normotensive** | **Screen-detected HPT** |
| hsa-miR-novel-chr1_36178 | MYNO2414 | UCCAGC | 17 | CUCCAGCCUGGGCAACA | 35.45 | 24.25 | - |
| hsa-miR-novel-chr15_18383 | MYNO1379 | GCUCCC | 22 | UGCUCCCCCUCCCUUCCUGGGA | 18.30 | - | 13.94 |

TPM - transcripts per million; HPT - hypertension
